# A Review of Carbohydrate Supplementation Approaches and Strategies for Optimizing Performance in Elite Long-Distance Endurance

**DOI:** 10.3390/nu17050918

**Published:** 2025-03-06

**Authors:** Wei Cao, Yong He, Ronghua Fu, Yiru Chen, Jiabei Yu, Zihong He

**Affiliations:** 1 School of Kinesiology, Shanghai University of Sport, Shanghai 200438, China; cw0616@126.com (W.C.);; 2 Exercise Biology Center, China Institute of Sport Science, Beijing 100061, China; 3 Beijing Research Institute of Sports Science, Beijing 100075, China

**Keywords:** endurance athletes, carbohydrates, gastrointestinal function, environmental factors, sports nutrition, Olympic Games

## Abstract

Carbohydrate supplementation is a common practice among endurance athletes participating in long-distance competitions. However, glycogen storage regulation, in-competition blood glucose levels, and their relationship with athletic performance are influenced by multiple factors. This review summarizes the recent research progress on carbohydrate supplementation, addressing its applications in the pre-, during-, and post-competition phases. It explores variables that influence the effectiveness of carbohydrate supplementation and provides a summary of strategies, based on six key aspects: carbohydrate properties, multi-nutrient interactions, gastrointestinal function, individual differences (such as age and gender), environmental conditions, and psychological factors. The combination of different types, ratios, and concentrations of carbohydrates has been demonstrated to enhance the efficiency of carbohydrate digestion and absorption. The synergistic combination of protein, sodium, and caffeine intake demonstrates enhanced efficacy in carbohydrate supplementation strategies. Gastrointestinal tolerance training for carbohydrate supplementation has been identified as an effective measure to alleviate gastrointestinal discomfort during high-dose carbohydrate intake. The adjustment of the carbohydrate-to-fat ratio and the type of carbohydrate intake has been found to mitigate the impact of gender and menstrual cycles on glycogen storage and substrate utilization. Modifying the timing of glycogen storage and regulating the concentration and temperature of carbohydrate solutions during competition have been demonstrated to facilitate coping with the elevated energy expenditure and metabolic substrate shift from fat to carbohydrates, triggered by a combination of environmental and psychological factors, including special environmental and climatic conditions (e.g., high altitude, high temperature, high humidity, and cold) and emotional states (e.g., pre-competition stress and anxiety during the competition). To achieve precise carbohydrate supplementation for athletes in major events under various competitive environments, it is necessary to quantitatively assess the effects of carbohydrate supplementation, supported by mechanistic studies. This can be achieved by utilizing wearable devices to monitor the entire competition, coupled with data collection technologies, such as high-throughput profiling. Furthermore, emerging data analytics techniques, such as machine learning and causal inference, should be leveraged to refine supplementation strategies.

## 1. Introduction

**Figure 1:**
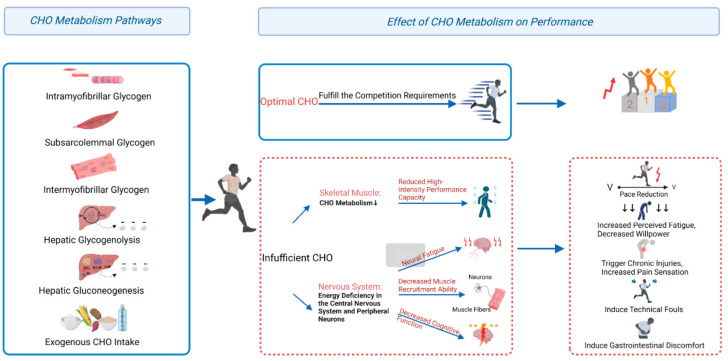
Carbohydrate supplementation enhances the performance of elite long-distance endurance athletes in competition.

Long-distance endurance events, defined as those lasting over 60 min, have consistently been a prominent component of the Summer Olympic Games, with each edition of the Olympics awarding more than 10 gold medals in these events. A notable example is the 2024 Paris Olympics, where 11 gold medals were awarded, including 3 in race walking, 2 in marathon, 2 in road cycling, 2 in mountain biking, and 2 in the 10-km swimming event. Race pace and performance outcomes in long-distance endurance events are influenced by multiple factors, including the level of competition, environmental conditions, specialized abilities, pre-race physiological state, and energy metabolism throughout the event. Given that aerobic carbohydrate oxidation serves as the primary energy source for athletes during high-intensity events [1], insufficient glycogen storages prior to the race or inadequate exogenous carbohydrate supplementation during the event may impair the ability to meet the energy demands, potentially leading to a decline in race pace during in the later stages [2]. The carbohydrates serving as metabolic fuel during endurance events for aerobic ATP (Adenosine 5′-triphosphate) production are predominantly stored as glycogen in the skeletal muscles (muscle glycogen, 300–400 g) and liver (liver glycogen, 80–100 g), with a minor amount circulating as glucose in the blood (approximately 5 g). The muscle and liver glycogen together account for a mere 4% of the body’s total energy reserves, and glycogen depletion has been recognized as a significant impediment to endurance performance [3]. Furthermore, a substantial positive correlation has been observed between glycogen content in Type I muscle fibers and endurance performance [4]. A decline in muscle glycogen levels that falls to 100 mmol·kg^−1^ dry weight prior to exercise has been shown to result in a 20–50% decrease in performance at 80% of peak power intensity [5]. Moreover, a decrease in muscle glycogen wet weight concentration that drops to 70 mmol·kg^−1^ has been observed to hinder the ability of muscle cells to generate sufficient ATP to maintain the same exercise intensity. Additionally, a decline in liver glycogen to below 30% renders the body incapable of expeditiously mobilizing glucose to maintain blood glucose homeostasis (4.0–5.5 mmol·L^−1^), consequently leading to diminished peak power output and premature fatigue [6]. Blood glucose levels during exercise exhibit a positive correlation with pacing within intervals, and total carbohydrate intake demonstrates a positive correlation with overall pacing [7]. In exercise, when blood glucose levels decrease below 3.9 mmol·L^−1^, the brain’s motor control centers and the nervous system’s capacity to recruit muscle fibers are compromised [8]. The phenomenon of “hitting the wall” (HTW) during marathon running is believed to arise from glycogen depletion and an insufficiency of energy substrates in the circulatory system [9]. These studies highlight the crucial role of glycogen storage and blood glucose levels in endurance performance. As shown in Figure 1, adequate glycogen storages and blood glucose levels are necessary to meet the energy demands of high-intensity competition, delay or prevent neural fatigue, and thereby assist athletes in maintaining peak performance. Therefore, scientifically informed carbohydrate supplementation emerges as one of the most effective means of enhancing outcomes in long-distance endurance events, either directly or indirectly. However, it is imperative to acknowledge that numerous factors can influence the relationship between glycogen storage, blood glucose levels, and competition outcomes, resulting in the observed variability in performance despite the application of consistent carbohydrate supplementation approaches. Those phenomena are more pronounced among outstanding athletes in major events, such as the Olympic Games. The level of competition and environment conditions [10,11] may significantly impact athletes’ carbohydrate metabolism and competition performance. Therefore, in contrast to recent studies [2,12], this review specially focuses on analyzing the factors affecting carbohydrate supplementation effectiveness and further comprehensively summarizes the improvement strategies employed by top endurance athletes in major events.

This review presents an in-depth synthesis of carbohydrate supplementation approaches and strategies aimed at enhancing the performance of elite endurance athletes. A systematic search was conducted across PubMed, Google Scholar, and Web of Science, prioritizing recent publications while also incorporating foundational studies in the field. The search employed key terms such as ‘endurance athletes’, ‘carbohydrates’, ‘gastrointestinal function’, ‘environmental factors’, ‘sports nutrition’, ‘gender differences’, ‘glycemic response kinetics’, and ‘psychological condition’, among others. Through this methodology, this review not only covers the full scope of the topic but also provides valuable insights into the current state of knowledge, highlighting areas where further empirical investigation is needed.

## 2. Carbohydrate Supplementation Approaches

### 2.1. Pre-Competition

The process of digesting and assimilating consumed carbohydrates into muscle and liver tissue as glycogen stores requires a minimum of four hours [13]. Therefore, pre-competition carbohydrate supplementation can be categorized into three phases: (1) carbohydrate loading to maximize glycogen storage, (2) the final pre-competition meal, and (3) supplementation between the final meal and the start of the competition.

It has been established that for every gram of glycogen stored, approximately 2.7 g of water are retained, which results in an increase in body weight during the glycogen loading phase. This increase may, in turn, lead to a slight reduction in exercise efficiency. Therefore, pre-competition carbohydrate supplementation is closely linked to the competition duration [14]. For events with a duration of less than 90 min, the standard recommendation is to consume 6–12 g·kg^−1^ of carbohydrates during the 24 h prior to the competition, with the aim of restoring glycogen levels to their normal state. For prolonged events exceeding 90 min duration, a glycogen-loading strategy is recommended, initiated 36–48 h before competition with a daily carbohydrate intake of 10–12 g·kg^−1^ [15]. Additionally, for the athletes competing in afternoon or evening events, attention to their carbohydrate intake at breakfast should be paid. Research suggests that even if lunch provides sufficient carbohydrates, a low-carbohydrate breakfast may reduce liver glycogen storage, negatively affecting endurance performance [16].

Typically, elite long-distance endurance athletes prepare for glycogen loading 10 days before the major event. During the 7–10 days before the competition, they primarily consume natural foods rich in carbohydrates, with three meals per day, avoiding additional carbohydrate powders or gels. During non-specialized training sessions, athletes may also reduce or eliminate carbohydrate drinks. In the final week (1–6 days before the event), the athletes increase their carbohydrate intake, including carbohydrate-rich foods at each meal, and resume carbohydrate drinks during training, along with supplementation of carbohydrate powders or gels. To avoid gastrointestinal discomfort, the total amount of carbohydrate supplementation is usually increased gradually. It is important to note that the carbohydrate intake should not exceed 75 g in the final pre-race meal [14], which should be consumed at least 2 h before the competition [17]. During the period from the final pre-race meal to the start of the race, including the time spent traveling to the competition venue, warm-up, and waiting in the check-in area, athletes often choose to consume small amounts of habitual carbohydrate solutions to maintain stable blood glucose levels and optimal hydration. It is recommended not to consume high doses of carbohydrates 60 to 30 min before the race. During the wait in the check-in area, athletes may rinse their mouths with carbohydrate drinks.

Carbohydrate distribution within skeletal muscle varies, with glycogen stored in the subsarcolemmal, inter- and intra-myofibrillar compartments, which account for 5–15%, 75%, and 5–15% of total muscle glycogen, respectively. During exercise, glycogen utilization in the intra-myofibrillar and subsarcolemmal regions takes precedence over that in the inter-myofibrillar region. Glycogen loading increases subsarcolemmal glycogen above normal levels, thereby sparing intra-myofibrillar glycogen use and improving endurance performance [4]. The final pre-competition meal and small doses of carbohydrate supplementation before the start avoid premature glycogen utilization and large fluctuations in blood glucose before the competition. A sudden rise or excessively high levels of blood glucose can lead to increased insulin release, which may impair fat oxidation, cause hypoglycemia, or trigger rebound hypoglycemia during the competition [15]. Most studies suggest that athletes should begin the competition with a normal blood glucose range [18].

### 2.2. During Competition

For endurance events lasting longer than 150 min, athletes are generally advised to ingest carbohydrates at a rate of 60–90 g·h^−1^. For events lasting between 60 and 150 min, athletes may choose to rinse with carbohydrate drinks or consume carbohydrates at a rate of 30–60 g·h^−1^. In events lasting ≤60 min, rinsing with carbohydrate-containing drinks is typically recommended. The total amount, timing, and frequency of carbohydrate supplementation are primarily determined by factors such as endogenous glucose production, exogenous carbohydrate oxidation rates, exercise intensity and duration, and environmental conditions [19].

Endogenous glucose production mainly occurs through hepatic glycogenolysis and gluconeogenesis, with a basal rate of approximately 0.1 g·kg^−1^·h^−1^. However, during periods of exercise at 55% of maximal output power and lactate threshold intensity, this rate can increase to 0.36 g·kg^−1^·h^−1^ and 0.48 g·kg^−1^·h^−1^, respectively [7]. The rate of exogenous carbohydrate oxidation exhibits a non-linear relationship with carbohydrate intake, suggesting that within a certain intake range, oxidation increases as the intake rises. However, beyond a certain intake threshold, the oxidation rate plateaus [20]. Thus, for endurance events exceeding 150 min, a carbohydrate intake rate greater than 90 g·h^−1^ may not lead to additional performance improvements [21]. Conversely, in circumstances where glycogen stores are insufficient, an intake rate exceeding 90 g·h^−1^ has been demonstrated to enhance endurance performance [22]. For events with a duration shorter than 150 min, there is an absence of evidence supporting the notion that a carbohydrate intake rate exceeding 60 g·h^−1^ results in additional performance enhancements [23]. Regarding the timing and frequency of supplementation, it is primarily determined by individual glucose kinetics and the available aid conditions during the competition. The time required for blood glucose levels to change following the intake of carbohydrate drinks is at least 15 min [24]. In a marathon, athletes can only retrieve their own drinks at fixed points every 5 km. In a 10 km swimming race, aid stations are provided every 1.6 km. In race walking events, aid stations are available every 1 km. Therefore, in major Olympic events, athletes should aim to supplement each time they pass an aid station.

During competition, as muscle glycogen levels decline, the continuous intake of exogenous carbohydrates has been shown to maintain glucose oxidation rates, preserve glycogen storage, and stabilize blood glucose levels. These effects collectively delay the onset of fatigue and reduce its severity [19]. Moreover, even in the glycogen depleted state, the supplementation of carbohydrates has been reported to enhance endurance performance [25]. When endogenous glucose production is sufficient to meet the energy demands of the event, the necessity of exogenous carbohydrate supplementation diminishes. In such cases, adequate carbohydrate intake can still effectively reduce the consumption of endogenous glucose, thus helping to maintain blood glucose balance while minimizing hepatic glycogen breakdown [7]. Without carbohydrate supplementation, plasma glucose contributes 19% to energy expenditure, whereas with high-concentration glucose intake during 2 h of exercise, this contribution increases to 42%. Therefore, blood glucose is considered the most important energy substrate in the later stages of prolonged exercise [26]. During intense exercise, hypoglycemia can occur in less than a minute, as the rate of glucose utilization can easily exceed the rate of hepatic glycogen breakdown and gluconeogenesis [1]. The most effective approach to carbohydrate supplementation during competition is to maximize exogenous carbohydrate intake without causing gastrointestinal discomfort. This approach ensures that carbohydrate oxidation does not become a limiting factor for performance. Maintaining stable blood glucose levels, or allowing them to gradually increase during the competition, is likely an ideal state for meeting the energy demands of long-duration endurance events and optimizing performance.

### 2.3. Post-Competition

After glycogen depletion, it requires at least 24 h for muscle glycogen and 11 h for liver glycogen to recover to pre-exercise levels [27]. The first 4 h post-competition, referred to as the “metabolic window”, are considered the most optimal time for carbohydrate supplementation at a rate of 1–1.2 g·kg^−1^·h^−1^ to promote glycogen recovery [19]. Within 24 h, a carbohydrate intake of 8–10 g·kg^−1^ is sufficient to restore glycogen stores [28]. Moreover, a sustained high carbohydrate intake (8 g·kg^−1^·day^−1^) over 36–48 h can lead to muscle glycogen supercompensation, effectively doubling muscle glycogen levels [29]. Furthermore, a positive correlation has been observed between an athlete’s skeletal muscle mass and their muscle glycogen storage capacity [29], suggesting that athletes with higher muscle mass should consume more carbohydrates to ensure adequate glycogen recovery.

A dose-dependent relationship has been observed between post-exercise carbohydrate intake and the recovery of endurance performance. Muscle glycogen recovery has been identified as a key determinant of subsequent endurance performance, while liver glycogen recovery has been shown to influence the timing of fatigue onset during later exercise bouts [30]. Consequently, the restoration of glycogen post-exercise is particularly crucial for athletes engaged in multiple events or back-to-back competitions. Glycogen restoration is initiated through gluconeogenesis at a rate of 1–2 mmol·kg^−1^·h^−1^. However, a high carbohydrate intake has been shown to accelerate glycogen resynthesis to 10 mmol·kg^−1^·h^−1^, persisting for up to 4 h [27]. The increase in plasma insulin concentrations following high carbohydrate intake post-exercise, in combination with enhanced insulin sensitivity, has been demonstrated to promote glucose transport into skeletal muscle [31].

## 3. Carbohydrate Supplementation Strategies

Glycogen storage, blood glucose levels, and their relationship with pacing and competition performance are influenced by a variety of factors. Even when the same carbohydrate supplementation protocol is followed, variations in performance occur due to differences in an athlete’s specialized abilities, physiological state, pre-competition conditions (such as training location), environmental factors (e.g., temperature and humidity), the level of competition, and pace tactics employed by opponents. As a result, athletes often exhibit divergent blood glucose dynamics during competition, with varied trends in blood glucose and pacing, thereby rendering the effects of carbohydrate supplementation uncertain.

### 3.1. Carbohydrate Supplementation Strategies Based on Carbohydrate Properties

Carbohydrates can be sourced from natural foods or processed supplements, which are available in various forms, such as solid bars, powders, gels, pastes, or liquids. The effects of different forms of the same carbohydrate on glycogen synthesis are generally similar. Therefore, gastrointestinal comfort is often the primary consideration when athletes select their carbohydrate sources [12]. However, it is crucial to note that the insulin dynamics, absorption pathways, transport carrier availability, gastric emptying rates, osmolarity, and gastrointestinal irritation vary across different carbohydrate types, leading to distinct absorption rates, oxidation rates, and supplementation effectiveness.

The classification of carbohydrates is based on their degree of hydrolysis and molecular weight, which categorizes them into four categories: monosaccharides, disaccharides, oligosaccharides, and polysaccharides. The blood glucose dynamics associated with these carbohydrates are influenced by hormones, such as insulin and glucagon. Monosaccharides and disaccharides have been shown to cause a rapid increase in blood glucose, while the blood glucose from oligosaccharides and polysaccharides occurs more gradually. Based on glycemic response velocity, foods are classified into high- or low-glycemic index (GI) categories. According to total carbohydrate content per serving, they are further categorized as high- or low-glycemic load (GL). Additionally, advancements in biotechnology have enabled the development of derivatives, such as isomaltose and trehalose, which, in comparison to glucose, may potentially reduce insulin responses and contribute to the maintenance of blood glucose stability [32].

The primary limitation for the absorption and transport of glucose and galactose is the sodium–glucose cotransporter 1 (SGLT1) on intestinal epithelial cells, while fructose is primarily transported by glucose transporter protein 5 (GLUT5). However, GLUT5 is less abundant than SGLT1, resulting in glucose being absorbed more rapidly and efficiently than fructose [33]. High doses of either glucose or fructose alone can cause gastrointestinal issues, such as diarrhea, abdominal pain, and bloating. However, the combination of glucose and fructose in supplementation has been shown to reduce or prevent gastrointestinal discomfort [19]. The concentration of carbohydrate drinks influences its absorption efficiency by modulating osmolarity and gastrointestinal function [34]. Biotechnological modifications of high-molecular-weight carbohydrates, such as highly branched cyclodextrin solutions, exhibit very low osmolarity, which has been found to accelerate gastric emptying and reduce gastrointestinal discomfort [32]. Similarly, gel forms of carbohydrates have also been demonstrated to speed up gastric emptying and facilitate absorption [35]. Therefore, athletes should strategically select the most suitable types of carbohydrates based on their physiological state and gastrointestinal function during different phases of competition. The precise adjustment of carbohydrate ratios and concentrations has the potential to minimize digestive discomfort, enhance absorption efficiency, and optimize glycogen storage and blood glucose homeostasis.

Prior to competition, carbohydrate loading with carbohydrate-fortified natural foods has been shown to enhance glycogen stores and provide essential nutrients and higher safety levels. To achieve adequate carbohydrate intake, elite athletes typically prioritize natural foods while also incorporating carbohydrate supplements. This approach is often complemented by multiple meals or snacks to further boost glycogen reserves. In the 48 h preceding the event, athletes are advised to consume familiar, easily digestible foods that are low in fat, dietary fiber, and GI, and high in carbohydrate content, to avoid gastrointestinal distress and prevent drastic fluctuations in blood glucose levels. After the final meal and before the race begins, the consumption of a low-GI beverage is particularly beneficial for maintaining energy metabolism during the competition [2].

During competitions, athletes commonly select mixed carbohydrate solutions to enhance the absorption and oxidation rate of exogenous carbohydrates. Additionally, certain long-distance endurance athletes choose whole foods, such as bananas, during the race. The maximum oxidation rate of glucose alone is around 1.0–1.2 g·min^−1^. However, when fructose is added, the oxidation rate can increase to approximately 1.5 g·min^−1^. Fructose has been shown to be particularly beneficial for liver glycogen replenishment [19]. The recommended ratio of fructose to glucose is typically 1:2, though some studies suggest that a ratio closer to 0.8:1 optimizes carbohydrate utilization efficiency, gastrointestinal comfort, and endurance performance [22].

It has been customary to recommend that athletes consume low-concentration or isotonic carbohydrate solutions during prolonged exercise, as these solutions are more easily absorbed and help maintain hydration [34]. A 6% glucose solution or an 8–10% glucose-fructose mixture is commonly advised. High-concentration solutions have been shown to slow gastric emptying. However, both the amount of carbohydrate delivered to the intestine and the oxidation rate of exogenous carbohydrates increase linearly with higher concentrations [34]. Due to the high intensity and gastrointestinal factors during long-distance endurance competitions, athletes typically consume smaller quantities of liquid (such as a mouthful) at each carbohydrate intake point. To meet their energy and carbohydrate demands, some athletes opt for higher-concentration solutions, even those exceeding 200 g·L^−1^, with optimal performance outcomes. Further research is needed to investigate this dose-response relationship and its effects on gastrointestinal function.

Additionally, carbohydrate supplementation during competition must consider the specific conditions of the event. To minimize errors, it is advisable to simplify the options available at aid stations, ideally combining carbohydrate and electrolyte solutions. However, athletes who cannot tolerate mixed carbohydrate–salt solutions should be allowed to maintain their preferred supplementation habits. In the post-competition phase, it is recommended to prioritize high-GI natural foods and various types of carbohydrate supplements. These have been shown to stimulate a stronger insulin response, which, in turn, promotes glycogen synthesis and enhances subsequent endurance performance [19].

### 3.2. Carbohydrate Supplementation Strategies Based on the Synergistic Enhancement Benefits of Protein, Sodium, and Caffeine

The combination of moderate amounts of protein, sodium, and caffeine in carbohydrate supplementation has been shown to have a synergistic effect, promoting carbohydrate metabolism, aiding functional recovery, reducing muscle damage, and improving endurance performance. Pre-competition supplementation with protein has been found to mitigate the insulin response triggered by substantial carbohydrate intake, which can impede fat mobilization and promote carbohydrate utilization. This approach has been observed to prevent the onset of premature fatigue, reactive or rebound hypoglycemia, and promote fat oxidation [18]. One study indicated that athletes engaged in 60 min of exercise at 70% VO_2max_, followed by 80% VO_2max,_ until reaching exhaustion. The athletes were given 1.2 g·kg^−1^ of carbohydrates, and either 0.4 g·kg^−1^ of protein 30 min before or at the start of the high-intensity portion. This supplementation reduced levels of alanine aminotransferase, aspartate aminotransferase, and creatine kinase 24 h after exercise [35]. Furthermore, in-competition supplementation with 0.25 g·kg^−1^·h^−1^ of protein has been shown to effectively mitigate fatigue and muscle damage [36]. Following exhaustive exercise, which resulted in a decline of muscle glycogen to 125 mmol·kg^−1^, the effects of two different nutritional interventions were examined. The first intervention involved the administration of 1.2 g·kg^−1^·h^−1^ of carbohydrates, while the second intervention consisted of a combination of carbohydrates (0.8 g·kg^−1^·h^−1^) and protein (0.4 g·kg^−1^·h^−1^). The latter group exhibited an 8.4-min increase in time to exhaustion during a 5-h cycling test compared to the carbohydrate-only group [37]. When carbohydrate intake is below 0.8 g·kg^−1^·h^−1^ post-exercise, adding protein (0.2–0.4 g·kg^−1^·h^−1^) can enhance the insulin response, promoting faster glycogen recovery [36]. However, when carbohydrate intake is sufficient, the increasing protein intake does not further improve endurance performance [30]. Given that many long-distance endurance athletes experience gastrointestinal discomfort, pre-competition carbohydrate loading often includes easily digestible, high-quality protein. During the 48 h leading up to the event, protein intake should be moderately reduced. During competition, given the increase in solution viscosity associated with the addition of protein powder, adjustments should be made based on race conditions, such as temperature and the athlete’s preferences. Immediately after the competition, protein supplementation (0.2–0.4 g·kg^−1^ body weight) can be calculated based on the athlete’s weight and either mixed with carbohydrates or consumed separately, according to the athlete’s habits.

Sodium, a fundamental electrolyte in the body, plays an essential role in maintaining extracellular volume, osmotic pressure, and gastrointestinal function [38]. Glucose absorption in the intestines is facilitated by co-transport with sodium, and the ingestion of sodium at concentrations ranging from 30 to 50 mmol·L^−1^ has been shown to enhance glucose uptake and utilization [39]. However, excessive sweating in high-temperature environments can disrupt this balance, leading to muscle cramps and hyponatremia. Consequently, athletes have been recommended to supplement sodium either prior to or during competition. Electrolyte salts are typically consumed in combination with carbohydrate solutions or separately. Symptoms such as vomiting, weakness, and muscle cramps during competitions may indicate hyponatremia. On the other hand, excessive sodium intake can lead to hypernatremia, which can cause similar issues and impair performance. Therefore, monitoring sodium loss through sweating during competition enables athletes to adjust their sodium intake in real time, thereby minimizing the risk of both hyponatremia and hypernatremia, while achieving the synergistic effects of combined sodium and carbohydrate supplementation. Additionally, some athletes have reported experiencing gastrointestinal discomfort, such as diarrhea, when consuming electrolyte salts, highlighting the importance of individualizing sodium supplementation and “training” in advance.

Furthermore, the integrated combination of nutrient supplementation has been shown to enhance the effects of carbohydrate intake. Caffeine, a widely used ergogenic aid among endurance athletes [40], has been found to promote fat breakdown and oxidation while reducing muscle glycogen depletion when ingesting 6–9 mg·kg^−1^ of caffeine 60 min before exercise [3]. In the 4-h recovery period following exhaustion, the combination of carbohydrates (1.2 g·kg^−1^) and caffeine (8 mg·kg^−1^) has been shown to accelerate muscle glycogen recovery [41]. However, the effects of caffeine are influenced by genetic factors, with athletes metabolizing caffeine at different rates depending on their genotype [40]. Some athletes may experience adverse effects, such as insomnia or dehydration, which can negatively impact performance. Therefore, the decision to use caffeine, as well as the appropriate dosage and timing, should be tested and refined during training before being applied in competition. Additionally, while caffeine may reduce the perception of fatigue, it does not eliminate fatigue itself. In other words, athletes may feel less fatigued after caffeine intake, but the underlying fatigue remains. For athletes who benefit from caffeine, it is essential to implement enhanced recovery protocols following training or competition. Furthermore, the tolerance to caffeine can vary among athletes, particularly during periods of pre-competition anxiety, which can exacerbate adverse effects, such as insomnia. Consequently, the dosage of caffeine should be adjusted based on the athlete’s training cycle, caffeine half-life, and emotional state prior to competition [42].

### 3.3. Carbohydrate Supplementation Strategies Based on Enhancing Gastrointestinal Function

Gastric emptying and intestinal absorption capacity are key determinants of glucose uptake and utilization. Gastrointestinal discomfort, however, significantly limits the oxidation rates of exogenous glucose [43]. Exercise-induced gastrointestinal syndrome (EIGS) is commonly observed in endurance athletes, with an incidence ranging from 30% to 90% [44,45]. The high prevalence of EIGS among endurance athletes is attributed to multiple factors, such as intense training, high-dose carbohydrate supplementation, elevated temperatures, and dehydration [46]. In major events like the Tokyo 2021 and Paris 2024 Olympics, many long-distance endurance athletes experienced gastrointestinal distress, including symptoms like vomiting and abdominal pain, which either led to race withdrawals or forced them to slow down to finish the event.

The etiology of gastrointestinal distress is multifactorial, encompassing factors such as insufficient athlete conditioning to sustain high race speeds or tactics. Additional contributing elements include insomnia, elevated stress levels, and discomfort from environmental factors (e.g., temperature) upon arrival at the competition venue. A rapid escalation of carbohydrate intake rates during competition, combined with sports beverages exceeding recommended carbohydrate concentrations and maintained at suboptimal temperatures, alongside elevated environmental heat and humidity levels during the event, may collectively contribute to gastrointestinal functional disturbances through synergistic physiological stressors. Collectively, these factors increase the likelihood and severity of gastrointestinal disturbances. It is noteworthy that the health status of athletes in the 10 days leading up to competition, particularly if they are experiencing diarrhea, can serve as a significant risk factor for gastrointestinal issues during the event [47]. Consequently, disparities in gastrointestinal function play a critical role in determining the effectiveness of carbohydrate supplementation.

**Figure 2:**
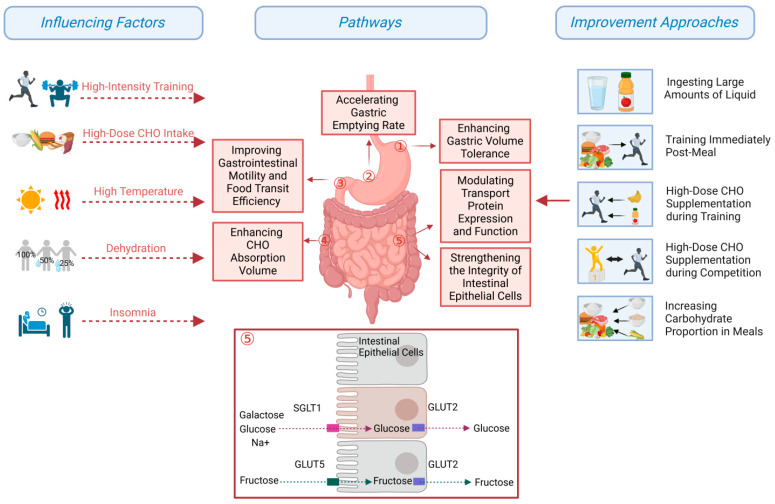
Gastrointestinal function improvement approaches based on influencing factors in elite long-distance endurance athletes.

The repeated intake of high carbohydrates before and during training has been demonstrated to condition the gastrointestinal system, improving its function, increasing tolerance, and reducing the incidence and severity of EIGS. These effects may, in turn, offer potential performance benefits [48]. On the one hand, high carbohydrate intake enhances gastric pressure adaptation, promoting more efficient gastric emptying. On the other hand, it has been observed to boost intestinal motility and transport, further optimizing overall gastrointestinal function [49]. Moreover, gastrointestinal tolerance training, which stimulates glucose transporter activity (such as SGLT1 and GLUT5), has the potential to augment the absorption capacity [49]. A study [50] assessed the impact of gastrointestinal tolerance training on endurance running performance. The study involved ten sessions of training over a two-week period, during which participants ran at 60% VO_2max_ for 60 min while ingesting a gel containing 30 g of carbohydrates at 0, 20, and 40 min. The results showed a significant reduction in gastrointestinal discomfort and a noticeable improvement in endurance performance. Thus, a scientifically designed “gastrointestinal function training” regimen emerges as an effective strategy for maximizing the efficacy of carbohydrate supplementation (Figure 2).

### 3.4. Carbohydrate Supplementation Strategies Based on Individual Differences in Age and Gender

Athletes exhibit considerable individual variation in their ability to digest and absorb carbohydrates, their gastrointestinal responses, and blood glucose kinetics. Moreover, substantial interindividual differences in glycogen storage have been documented, with muscle glycogen content ranging from 300 to 1000 mmol·kg^−1^, and liver glycogen content varying from 270 to 400 mmol·kg^−1^ [6]. These findings emphasize the need for personalized carbohydrate supplementation strategies during both training and competition. For instance, one study developed a model to provide individualized pacing and carbohydrate intake recommendations for ultramarathon runners, incorporating factors such as altitude, terrain, distance, and the athlete’s body weight, cardiovascular health, and psychological state [51]. Another study employed continuous glucose monitoring (CGM) to investigate the relationship between dynamic blood glucose levels and pacing during competition, further highlighting the importance of individualized carbohydrate supplementation [2,7]. Furthermore, endurance athletes tend to reach their peak performance at older ages and sustain a longer athletic career, leading to a broader age range in competitive events [52]. Age influences athletic performance in various ways, with factors such as changes in basal metabolic rate [53], mitochondrial capacity and insulin sensitivity [54], appetite and glucose homeostasis [54], and nutrition habits and beliefs [55], all serving as important determinants of energy expenditure and carbohydrate metabolism. Consequently, different age stages, from adolescence to adulthood, should be considered essential when developing personalized carbohydrate supplementation strategies for athletes. However, research on this area remains limited.

Gender-based differences in carbohydrate metabolism and supplementation efficacy have been well-documented. Female athletes, compared to males, benefit from the effects of estrogen, particularly 17-β estradiol, which promotes fat breakdown and enhances the availability of fatty acids [56]. During exercise, women tend to exhibit greater lipid oxidation and relatively lower glycogen utilization. Additionally, the depletion of intramyocellular glycogen during exercise has been found to be significantly higher in males compared with females [57]. Hormonal fluctuations throughout the menstrual cycle, including changes in 17-β estradiol and progesterone, influence the balance between carbohydrate and fat utilization during both rest and exercise [58], which may contribute to the increased difficulty women experience in glycogen storage during the follicular phase. Additionally, these hormonal fluctuations affect appetite and food preferences [59]. Menstrual irregularities are more common among long-distance endurance athletes [60], whereas athletes with regular menstrual cycles demonstrate a greater ability to maintain blood glucose levels during prolonged exercise [61].

Given the impact of gender differences and hormonal fluctuations on carbohydrate metabolism, athletes require periodized nutritional protocols to address their carbohydrate needs across all phases of their menstrual cycle [58]. Especially during the follicular phase, female athletes typically enhance glycogen stores through multiple smaller meals. Athletes with menstrual irregularities may gain an advantage from increased intake of high-glycemic foods to counteract these effects and improve performance. Further research is required to clarify how hormonal variations during the menstrual cycle impact carbohydrate metabolism in female athletes, which could facilitate the development of more tailored supplementation strategies.

Moreover, approximately 80% of long-distance endurance athletes exhibit at least one symptom of relative energy deficiency in sport (RED-S) [62]. Insufficient carbohydrate intake is a primary contributor to RED-S [63]. The negative energy balance associated with RED-S further reduces the availability of glucose for circulation, thereby decreasing exogenous glucose oxidation rates [19] and endurance performance [64]. However, excessive carbohydrate intake can lead to exercise-induced gastrointestinal syndrome (EIGS), which also impairs athletic performance [48]. Therefore, individualized carbohydrate intake, in line with recommended guidelines, is crucial for preventing both RED-S and EIGS, while promoting optimal exogenous glucose oxidation.

### 3.5. Carbohydrate Supplementation Strategies in Extreme Environments

**Figure 3:**
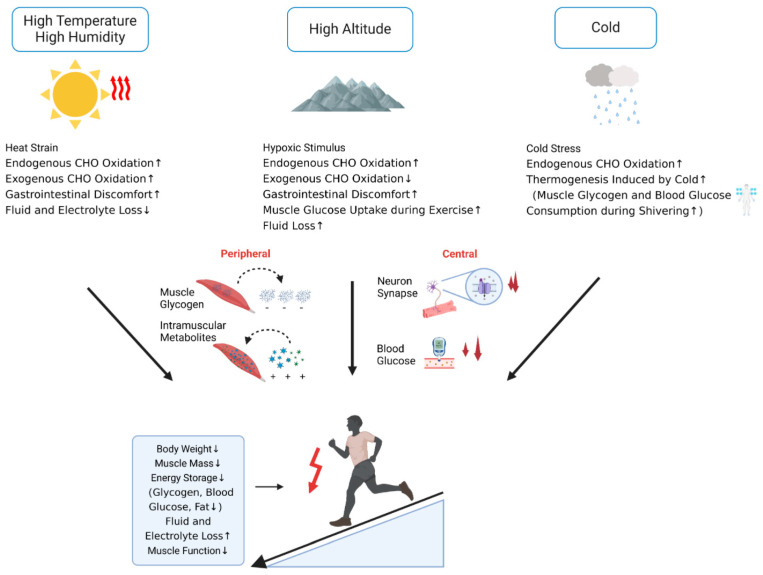
Direct (solid arrows) and indirect (dashed arrows) effects of environmental conditions on carbohydrate metabolism in elite long-distance endurance athletes. Arrows indicate: ↑ Increased physiological demand/consumption; ↓ Reduced metabolic efficiency/storage.

Endurance athletes often compete in extreme environments, such as the high-temperature and high-humidity conditions at the 2019 IAAF World Athletics Championships in Doha, the 2021 Tokyo Olympics, and the 2023 World Athletics Championships in Budapest. Other notable events include the 2015 IAAF World Cross Country Championships in Guiyang, China (elevation 1275 m), the 2017 IAAF World Cross Country Championships in Kampala, Uganda (elevation 1210 m), and the Russian marathon held in −53 °C conditions. Environmental factors, including high altitude, heat, humidity, and extreme cold, have been shown to alter an athlete’s basal metabolic rate, endogenous and exogenous glucose oxidation rates, sweat rate, gastrointestinal function, blood volume, appetite, and food preferences. These changes may lead to fatigue, injury, and disruptions in energy metabolism and physiological function, ultimately influencing athletic performance [46] (Figure 3).

#### 3.5.1. Carbohydrate Supplementation Strategies in High-Altitude Environments

High-altitude training is a widely adopted practice among endurance athletes, typically incorporated into both winter and summer cycles, as well as in the period leading up to competitions. However, this approach can be a double-edged sword. Research indicates that high-altitude training leads to an increase in resting metabolic rate, an elevation in endogenous carbohydrate oxidation, and a reduction in exogenous carbohydrate oxidation [46]. Hypoxia at high altitudes has been shown to exacerbate gastrointestinal issues and accelerate both peripheral and central fatigue [65]. Peripheral fatigue primarily stems from muscle glycogen depletion and the accumulation of intramuscular metabolites, while central fatigue is typically driven by fluctuations in blood glucose levels, neurotransmitter concentrations, and reduced central motor drive due to arterial hypoxemia [65]. The ingestion of carbohydrates, which helps to reduce muscle glycogen depletion and maintain blood glucose levels, has been demonstrated to mitigate or delay the onset of peripheral and central fatigue induced by hypoxia and/or training [65]. Nevertheless, athletes often experience reduced appetite and a preference for high-fat foods during high-altitude training [66]. For ultramarathon runners, the ingestion of 120 g·h^−^ [1] of carbohydrates has been shown to postpone the onset of fatigue during a one-day race at high altitude. However, many athletes struggle to consume even the recommended 90 g·h^−1^ due to gastrointestinal discomfort or decreased appetite [67]. Adaptation to high-altitude and hypoxic conditions also exhibits significant gender disparities, with male athletes requiring higher carbohydrate intake to prevent the shift in metabolic substrate use from fat to carbohydrates [68]. Moreover, when training at the same altitude, athletes who are native to high altitudes should be distinguished from those who come from lowland regions.

During high-altitude competitions or the final week of altitude training prior to a race, glycogen storage challenges become multifaceted. These difficulties include increased energy expenditure, reduced appetite, decreased willingness to consume carbohydrates, a shift in metabolic substrates from fat to carbohydrates, elevated endogenous carbohydrate utilization, and exacerbated gastrointestinal issues. Consequently, pre-competition training at high altitudes, particularly when accompanied by gastrointestinal discomfort, compromised immunity, or injury, may benefit from approaches aimed at improving gastrointestinal function, optimizing glycogen storage timing, and increasing dietary carbohydrate intake. Further research is necessary to determine the optimal timing for glycogen storage, the ideal types and proportions of carbohydrates, and the mechanisms through which altitude influences supplementation effectiveness. These insights could help refine carbohydrate supplementation strategies and enhance performance outcomes.

#### 3.5.2. Carbohydrate Supplementation Strategies in Hot and Humid Environments

The ideal environmental conditions for endurance events are typically found when the ambient temperature ranges from 10 °C to 17.5 °C, or when the Wet Bulb Globe Temperature (WBGT) is between 7.5 °C and 15 °C. For marathon runners, a WBGT of 7.5 °C is considered optimal, with each 1 °C deviation resulting in an estimated 0.1% decline in performance. In the 20 km race walk, the best performance is observed at a WBGT of 12.5 °C, while every 1 °C increase leads to approximately a 0.4% decline in race results. As a result, the 20 km race walk is more heat-sensitive than other endurance events [69]. In practice, nearly one-quarter of endurance competitions are held in environments with moderately hot (WBGT 18.5–23 °C, ~18%), highly hot (WBGT 23.1–28 °C, ~7%), or extremely hot (WBGT > 28 °C, ~1%) conditions [69]. During such competitions, athletes may reach a “critical temperature threshold”, where a core temperature surpasses a certain level or remains elevated for a limited period, leading to a marked decline in performance, resulting in slower pacing or even race withdrawal [70]. At low speeds (3.2–9.7 km·h^−1^), core temperature has been shown to rise linearly with speed and exercise duration [71]. Therefore, at high speeds, endurance athletes are likely to experience a more rapid and pronounced escalation in core temperature.

In hot environments, an increase in core temperature and subsequent decline in athletic performance can be attributed to two crucial pathways. The first involves heat-induced conditions, such as “heat stroke”, which can arise under such circumstances [70]. Training and competition in environments with a WBGT between 23.1 °C and 28 °C, or exceeding 28 °C, present a moderate-to-high risk of heat stroke [69]. The second pathway entails a shift in energy substrate utilization, where high temperatures lead to a reduction in fat oxidation, an increase in endogenous carbohydrate oxidation, and a decrease in exogenous carbohydrate oxidation [46]. For example, at an outdoor temperature of 35.4 °C, when athletes exercise at 55% VO_2max_ for 90 min, exogenous carbohydrate oxidation rates decrease by 10%, while muscle glycogen oxidation rates increase by 25% [72]. In another study, cycling at 95% maximum heart rate for 90 min at an external temperature of 33 °C led to a 19% decrease in total carbohydrate oxidation, accompanied by a 17% reduction in average power output. This decline was positively correlated with the external temperature [73]. Furthermore, high humidity has been shown to exacerbate or compound the body’s responses to heat during competition, increasing discomfort among athletes [74]. Consequently, in hot and humid conditions, multiple factors converge to amplify the demand for endogenous carbohydrates.

Overall, competing in hot and humid environments significantly increases the consumption of carbohydrates and the rate of perspiration. Furthermore, the combination of high heat and humidity worsens gastrointestinal discomfort, dehydration, and sodium loss, leading to hyponatremia or hypernatremia. These conditions may result in vomiting or diarrhea, which reduces the effective absorption of carbohydrates, making carbohydrate supplementation more challenging. To optimize performance in such hot and humid conditions, athletes should aim to maximize their carbohydrate stores during the week leading up to the competition, provided their gastrointestinal tolerance permits. Additionally, athletes should increase their water intake 2–3 h before the competition to ensure proper hydration, which will help maintain adequate carbohydrate stores and good hydration status, supporting sustained athletic performance while minimizing carbohydrate depletion due to dehydration. During the event, athletes commonly consume carbohydrate drinks stored in ice-water mixtures, which both provide carbohydrate supplementation and help reduce core temperature. However, ice water may exacerbate pre-existing gastrointestinal discomfort. Therefore, athletes should practice this strategy in advance to balance the benefits and potential drawbacks.

#### 3.5.3. Carbohydrate Supplementation Strategies in Cold Environments

Approximately 27% of endurance competitions are held in cold environments (i.e., with temperature ≤ 10 °C) [69]. For instance, during the 2024 Italian Modugno Marathon Race Walk Mixed Relay, temperatures ranged from 3 °C to 8 °C. Similarly, at the first leg of the 2024 Jiangsu Taicang World Athletics Race Walking Challenge and Paris Olympic Selection Trials, temperatures during athlete warm-ups on March 3 and March 9 were around 1 °C and 3 °C, respectively, with race start temperatures between 3 °C and 9 °C. In long-distance endurance events, every 1 °C decrease from the optimal WBGT (7.5 °C for marathons, 15 °C for 50 km race walks, and 12.5 °C for 20 km race walks) is associated with a 0.3% ± 0.2% decline in performance [69].

Cold exposure has been shown to elicit a range of metabolic responses through the activation of the sympathetic nervous system and neurohormonal pathways, such as the renin–angiotensin–aldosterone system. These responses include enhanced shivering and non-shivering thermogenesis, along with alterations in energy substrate utilization. For example, immersion in 18 °C water for 90 min (or when core temperature drops to 35.5 °C) leads to a 3.5-fold rise in resting metabolic rate, while muscle glycogen and blood glucose levels decline by 20% [75]. Furthermore, reducing room temperature from 29 °C to 10 °C results in a 2.5-fold increase in resting metabolic rate, accompanied by a 6-fold increase in endogenous carbohydrate oxidation rates [76]. In addition, at temperatures between 18 °C and 19 °C, shivering thermogenesis is observed to be twice as active compared to at 25 °C, and the administration of insulin may lead to hypoglycemia, with blood glucose levels dropping below 2.5 mmol·L^−1^. Following hypoglycemia, shivering thermogenesis is completely suppressed, but it recovers once blood glucose levels are restored [77]. These findings suggest that during increased thermogenesis in cold environments, the primary energy substrates are muscle glycogen and blood glucose. Hypoglycemia is also considered an early warning sign that the body may be approaching hypothermia (below 35 °C).

During high-intensity endurance competitions, carbohydrates serve as primary energy substrates. In cold environments, the combination of high-intensity exertion and the subsequent depletion of muscle glycogen and lowering blood glucose levels elevates the risk of premature glycogen exhaustion and hypoglycemia. Moreover, cold environments reduce nerve conduction velocity [78], and when coupled with the effects of hypoglycemia on the nervous system [8], neuromuscular control is further impaired. Those factors may contribute to an increased incidence of injuries, more frequent disqualifications, and diminished performance during competitions held in cold conditions. Studies have demonstrated a linear negative correlation between maximum power output and VO_2max_ with core temperature (37.5–34.9 °C) and muscle temperature (38.0–35.1 °C). Quantitatively, experimental data delineate a 1 °C temperature decline correlating with a 20% reduction in maximum power output and a 5–6% decrease in VO_2max_ [70].

To enhance performance in cold environments, it is essential to maintain adequate total energy and carbohydrate intake, as these support glycogen storage and blood glucose homeostasis, which are crucial for endurance athletes. For athletes experiencing gastrointestinal discomfort, consuming carbohydrate solutions prepared with warm water during the event can alleviate gastrointestinal issues and increase carbohydrate absorption. Additionally, real-time blood glucose monitoring, in conjunction with adjustments to the carbohydrate beverage concentration, can help optimize blood glucose level and meet the energy demands of the competition.

### 3.6. Carbohydrate Supplementation Strategies Under Competitive Anxiety

In major competitions, such as the Olympic Games, pre-competition anxiety and nervousness are unavoidable [79], and those emotions tend to intensify as the event date approaches [43]. The level of nervousness experienced prior to competition has been demonstrated to affect an athlete’s appetite and blood glucose levels. Long-distance endurance major events typically involve many highly skilled competitors. For instance, in the 2023 World Athletics Championships, 50 athletes participated in the men’s 20 km race walk. Following the starting gun, the leading group was large, with numerous competitors employing high-paced tactics. The top three finishers recorded times of 1:17:32, 1:17:39, and 1:17:47, respectively, while the 4th to 10th place finishers had times between 1:18:03 and 1:18:59, and the 11th to 18th placed athletes finished between 1:19:01 and 1:19:55. The competition was intense, and, therefore, the pressure and anxiety during the race were also inevitable.

When athletes experience anxiety, their stress-response metabolic pathways are activated. The sympathetic nervous system is the primary pathway involved, leading to the release of catecholamines, such as norepinephrine and epinephrine, from the adrenal medulla. These catecholamines regulate a series of stress responses through the sympathetic–adrenal–medullary axis [80]. In the context of normal or elevated glycogen levels, the injection of epinephrine (20 μg/100 g body weight) over a period of 3 h has been observed to result in a significant decrease in muscle glycogen levels, exceeding 30% [81]. Conversely, catecholamine injections (20 μg/100 g body weight) over a duration of 2 h have resulted in a 41% reduction in muscle glycogen [82].

The second pathway involves the activation of the hypothalamic–pituitary–adrenal (HPA) axis, which triggers the release of the corticotropin-releasing hormone (CRH). This, in turn, induces the secretion of the adrenocorticotropic hormone (ACTH) from the pituitary gland and stimulates the release of glucocorticoids, such as cortisol [83]. Cortisol activates glycogen metabolism in both the liver and muscles by stimulating glycogen phosphorylase, glucagon, and epinephrine [84]. Furthermore, emotional stress has been demonstrated to exacerbate gastrointestinal discomfort [43].

Athletes participating in long-distance endurance events often experience a decrease in appetite, unexplained diarrhea, and insomnia during pre-race training or upon arrival at the competition venue. Those symptoms suggest that heightened anxiety and emotional stress during the competition may increase glycogen consumption while impairing the effective absorption of carbohydrates due to gastrointestinal discomfort, further diminishing glycogen reserves. Consequently, athletes experiencing elevated anxiety levels due to high-intensity training and various pressures may benefit from an advanced carbohydrate loading period and an increased total carbohydrate intake, in combination with other forms of supplementation. This strategy helps optimize endogenous glycogen stores and alleviate anxiety. In cases where gastrointestinal distress limits carbohydrate intake during competition, increasing the concentration of the carbohydrate solution to boost exogenous carbohydrate intake may effectively mitigate early or excessive glycogen depletion caused by anxiety. This approach ensures an adequate energy supply during the competition.

## 4. Limitations and Future Research Directions

Scientifically guided carbohydrate supplementation is one of the key strategies for ensuring optimal performance in elite endurance athletes. However, achieving precise carbohydrate supplementation requires further investigation into several unresolved issues.

Firstly, there is a lack of integrated studies examining the synergistic interactions between the core factors that influence the performance of elite endurance athletes. Additionally, current methods for glycogen testing, such as Bergström muscle biopsy [85], magnetic resonance spectroscopy (using 13 C-glucose via ingestion or intravenous injection) [22], and musculoskeletal high-frequency ultrasound [86], face challenges in enabling rapid, accurate, and real-time monitoring of glycogen storage and depletion during training and competition. This limitation significantly hinders the study of carbohydrate metabolism.

Secondly, previous studies have primarily identified the effects of factors, such as altitude, temperature, gastrointestinal function, and the menstrual cycle, on carbohydrate metabolism. However, conclusive evidence regarding the causal and dose-response relationships between these factors and carbohydrate metabolism remains lacking. Additionally, predictive models for glycogen deficiency are notably lacking.

Thirdly, to avoid the adverse effects associated with excessive carbohydrate supplementation, recent strategies, such as the “sleep low, train low”, approach have gained attention. Building on this, the “glycogen threshold” hypothesis has been proposed, suggesting that the existence of an optimal glycogen threshold that can meet the energy demands of training and competition while simultaneously activating molecular pathways involved in endurance adaptation [87]. However, empirical research to support this hypothesis remains limited.

**Figure 4:**
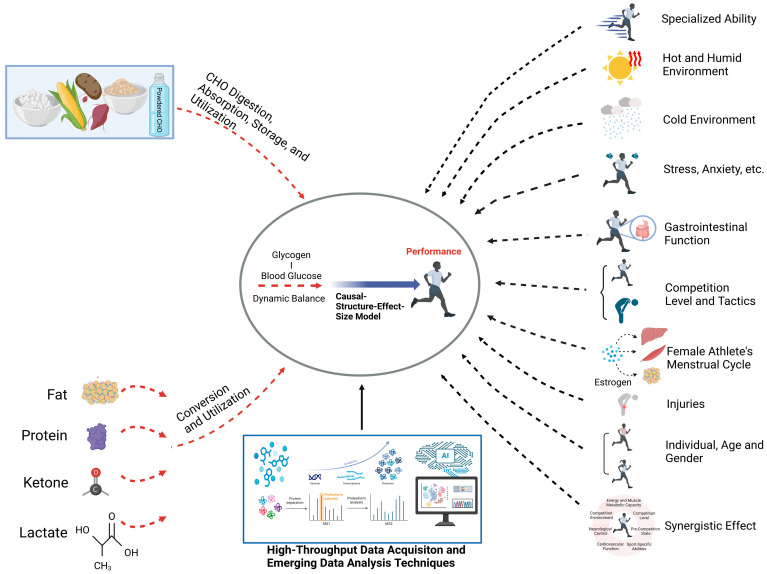
Future directions in precision carbohydrate supplementation for elite long-distance endurance athletes.

Thus, as shown in Figure 4, achieving precise carbohydrate supplementation for long-distance endurance athletes requires further experimental research in several key areas:

(1) The energy requirements and carbohydrate metabolism characteristics under different competition levels, environments, specialized abilities, and athletic states, along with corresponding carbohydrate supplementation strategies.
(2) The capacity for carbohydrate-based energy provision during major competitions, the optimal glycogen threshold, the appropriate blood glucose range, and real-time monitoring methods for glycogen storage and depletion, all of which are crucial for enhancing endurance performance.
(3) The causal relationships between athletes’ specialized abilities, carbohydrate metabolism, and gastrointestinal function, and the development of predictive models for competition performance based on these relationships.
(4) The molecular mechanisms of carbohydrate transport, storage, and utilization, as well as their interaction with other energy substrates (e.g., fats, proteins, ketones, and lactate) in energy metabolism. This includes precise glycogen storage patterns in subsarcolemmal, inter-myofibrillar, and intra-myofibrillar regions.

Of particular importance is the introduction of wearable devices and high-throughput omics data collection technologies during competitions. The integration of emerging data analysis techniques (including machine learning and causal inference) with traditional statistical methods is recommended. This approach will facilitate the quantitative identification of supplementation effects and the causal modeling of common mechanisms, ultimately laying the foundation for precise carbohydrate supplementation tailored to athletes’ specialized abilities, athletic states, and competition environments.

## 5. Conclusions

Glycogen storage and exogenous carbohydrate supplementation are crucial for optimizing competition performance in long-distance endurance athletes. To maximize the effectiveness of carbohydrate supplementation, it is essential to consider factors such as carbohydrate properties, synergistic nutrients (e.g., protein, sodium, and caffeine), gastrointestinal comfort, the individual, age and gender differences, environmental conditions, and psychological factors like competitive anxiety, and to develop appropriate strategies to address those issues. Personalizing carbohydrate supplementation strategies to individuals’ needs can improve energy availability, delay fatigue, and enhance the overall efficacy of the supplementation. By implementing these tailored strategies, athletes can achieve improved performance, accelerate recovery, and attain better outcomes in both training and competitions.

## Abbreviations

The following abbreviations are used in this manuscript:

HTW	Hitting the wall
SGLT1	Sodium–glucose cotransporter 1
GLUT5	Glucose transporter protein 5
GI	Glycemic index
EIGS	Exercise-induced gastrointestinal syndrome
CGM	Glucose monitoring
WGBT	Wet Bulb Globe Temperature
HPA	Hypothalamic–pituitary–adrenal
CRH	Corticotropin-releasing hormone
ACTH	Adrenocorticotropic hormone
ATP	Adenosine 5′-triphosphate
GL	Low glycemic load
